# Thromboelastography versus bleeding time for risk of bleeding post native kidney biopsy

**DOI:** 10.1080/0886022X.2019.1700805

**Published:** 2019-12-16

**Authors:** Amir Gal-Oz, Amitay Papushado, Ilya Kirgner, Shmuel Meirsdorf, Doron Schwartz, Idit Francesca Schwartz, Asia Zubkov, Ayelet Grupper

**Affiliations:** a ICU Department, Tel-Aviv Sourasky Medical Center and Sackler Faculty of Medicine, Tel-Aviv University, Tel-Aviv, Israel; b Department of Internal Medicine “B”, Tel-Aviv Sourasky Medical Center and Sackler Faculty of Medicine, Tel-Aviv University, Tel-Aviv, Israel; c Hematology Department, Tel-Aviv Sourasky Medical Center and Sackler Faculty of Medicine, Tel-Aviv University, Tel-Aviv, Israel; d Radiology Department, Tel-Aviv Sourasky Medical Center and Sackler Faculty of Medicine, Tel-Aviv University, Tel-Aviv, Israel; e Nephrology Department, Tel-Aviv Sourasky Medical Center and Sackler Faculty of Medicine, Tel-Aviv University, Tel-Aviv, Israel; f Pathology Department, Tel-Aviv Sourasky Medical Center and Sackler Faculty of Medicine, Tel-Aviv University, Tel-Aviv, Israel

**Keywords:** Thromboelastography, kidney biopsy, uremic bleeding, renal failure

## Abstract

**Introduction:**

The risk of bleeding has led to screening of the primary hemostasis before renal biopsy. A bleeding time test (BT) is considered standard practice, but reliance on this test is controversial and its benefits remain questionable. A possible alternative is thromboelastography (TEG). However, data regarding TEG in patients with renal dysfunction is limited.

**Objectives:**

To determine TEG abnormalities and their consequences in patients who underwent a native kidney biopsy.

**Methods:**

A retrospective study of 417 consecutive percutaneous native renal biopsies performed in our Center. If serum creatinine >1.5 mg/dL, the patient underwent either a BT test (period A, January 2015–31 December 2016) or TEG (period B, January 2017–August 2018). In patients with prolonged BT, or an abnormal low maximal amplitude (MA) parameter of TEG, or suspected clinical uremic thrombopathy, the use of desmopressin acetate (DDAVP) was considered.

**Results:**

Most biopsies (90.6%) were done by the same dedicated radiologist. Fifty-one patients had a BT test, which was normal in all tested patients. Seventy-one patients underwent TEG, and it was abnormal in 34 of them, most patients had combined abnormalities. The only parameter related to abnormal TEG was older age (Odds Ratio 1.21 [95% CI 1.09–2.38] *p* = 0.04 for abnormal Kinetics; OR 1.37 (1.05–1.96) *p* = 0.037 for abnormal MA). Twenty-six patients (6.23%) had bleeding complications. Risk of bleeding was significantly related to age (1.4 [1.11–7.48] *p* = 0.04), systolic blood pressure (1.85 [1.258–9.65] *p* = 0.02), and serum creatinine (1.21 [1.06–3.134] *p* = 0.048).

**Conclusions:**

TEG abnormalities in patients with renal dysfunction are variable and fail to predict bleeding during kidney biopsy. The decision to administer DDAVP as a preventive measure during these procedures should be based on clinical judgment only.

## Introduction

While percutaneous renal biopsies may be obtained to establish diagnosis, help determine therapy and ascertain the degree of active and chronic changes in the kidney [1], indications for performing such a procedure vary among nephrologists, determined largely by the presenting signs and symptoms [1–3].

Bleeding is the primary complication of renal biopsy [1,4]. Compared with biopsies from other sites, biopsy of the kidney has the greatest risk of post-procedure hemorrhage [5].

While abnormal platelet-endothelial interaction is associated with excessive bleeding in chronic renal failure, platelet dysfunction is the most significant factor that lies behind excessive bleeding [6]. This is due to both decreased platelet aggregation and impaired platelet adhesiveness, which result, at least in part, from intrinsic dysfunction of glycoprotein IIb/IIIa [7,8]. Although uremic patients may also suffer infrequently from factors related to reduced blood coagulation and thrombocytopenia, these symptoms are not considered to be major elements in the setting of uremic hemostatic defect [9].

The risk of bleeding has led to standard screening of the primary hemostasis before a renal biopsy is performed [10], although no strong evidence exists to support this practice. While a bleeding time (BT) test is considered to be standard practice for the assessment of platelet function in uremic patients [11], it requires technical expertise, has questionable reproducibility and accuracy, and poorly predicts clinical bleeding risks [12–15]. Although there are no randomized prospective studies evaluating the use of a BT test in the setting of a percutaneous renal biopsy, observational studies have demonstrated a higher bleeding complication rate in those patients with abnormal test results [16–21]. Other series, however, report no increased risk [22,23], so the value of this practice remains to be debated [22,24]. Platelet function analyzer-100 (PFA-100) test measures activated platelets and is more sensitive in comparison with the bleeding time for bleeding disorders. However, it was not found to be useful in predicting bleeding post kidney biopsy [25,26].

A possible alternative for the BT test is thromboelastography (TEG). TEG tests both platelet function and coagulation by assaying several parameters of clot formation dynamically in whole blood, thus providing a detailed, real-time assessment of a patient’s hemostasis profile [27,28]. TEG evaluates clotting in three distinct phases: clot initiation, clot propagation, and fibrinolysis.

TEG has been used successfully as a point-of-care test within surgical departments [29,30]. However, data assessment regarding TEG in patients with renal dysfunction is limited [31,32]. The aims of our study are to determine TEG abnormalities and their consequences in patients who underwent a native kidney biopsy.

## Materials and methods

### Patient population

Between January 2015 and September 2018, we retrospectively studied 417 consecutive percutaneous native renal biopsies performed in adults (>18 years old) in the Tel Aviv Sourasky Medical Center. For the purpose of this study, the time period from January 2015 to 31 December 2016 was defined as period A, and from January 2017 to August 2018 as period B.

All kidney biopsies were performed either by an experienced radiologist or nephrologist. In most cases the procedure was done by the same dedicated radiologist (S.M.), using real-time ultrasound imaging. Informed consent was obtained from all patients prior to the biopsy.

All patients underwent a thorough evaluation before biopsy including blood pressure measurements, prothrombin time (PT), activated partial thromboplastin time (aPTT), and complete blood count. If serum creatinine was higher than 1.5 mg/dL, the patient underwent further evaluation by either a BT test (period A) or TEG (period B). In patients with prolonged BT (>6 min) or an abnormal low maximal amplitude (MA) component of TEG (<50 mm), the use of desmopressin acetate (DDAVP) was considered (intravenously at a dose of 0.3 mcg/kg). The use of DDAVP was also considered in patients with suspected uremic thrombopathy in the absence of abnormal BT or TEG.

Non-steroidal anti-inflammatory drugs, antiplatelet agents and anticoagulants were discontinued 5 days before the biopsy.

### Kidney biopsy technique

The patient was placed in a prone position with a pillow under the abdomen. Percutaneous renal biopsy was performed under ultrasonic guidance with local anesthesia (2% lidocaine hydrochloride). A skin mark was made to identify where the biopsy needle would be inserted. The site was subsequently prepped and anesthetized. Under ultrasound guidance, a spinal needle was then used to locate the capsule of the kidney and to provide anesthesia for the biopsy needle tract. The choice of biopsy needle was left to the discretion of the radiologist, who usually used a 16-gauge needle, unless there were special considerations.

The procedure was usually performed on the left kidney, unless there were specific considerations to the contrary. The approach, determined by physician preference, was usually a cortical tangential approach. Biopsies were performed without immediate sample assessment by on-site pathology staff. A minimum of 3 cores were obtained during the procedure, based on the decision of the radiologist according to the cores obtained.

Outpatients were monitored 4–6 h after biopsy before discharge if they were hemodynamically stable and without pain. Patients were monitored closely following biopsy for signs or symptoms of complications, such as gross hematuria or hypotension. Vital signs were checked every 30 min during the first 2 h post biopsy and then every 2 h until the following day or until the patient was discharged. Blood count was measured at approximately 5–8 and 18–24 h after the procedure (only for admitted patients), and the lowest post biopsy hemoglobin level was recorded. The need for additional studies or treatment was determined by the attending nephrologist.

Bleeding complications were defined as the need for blood products for any reason; and/or decreased hemoglobin of >1.5 g/dL post biopsy; and/or invasive intervention (surgical or angiographic) during the following day.

Hypotension was defined as decreased blood pressure (systolic blood pressure >20 mmHg and/or diastolic >15 mmHg) during the post-biopsy day.

A normal platelet count was defined as 150–450 × 10^9^/L; normal PT was defined as 10.0–12.4 s; normal aPTT was defined as 25.0–34.0 s.

Glomerular filtration rate was estimated (eGFR) using the Chronic Kidney Disease Epidemiology Collaboration (CKD-EPI) creatinine equation, a 4-variable formula [33] adjusted for body surface area (Mosteller calculation).

### TEG test

The Thrombelastograph^®^ (TEG^®^) Hemostasis System (Haemoscope Corporation, Niles, IL, USA) equipped with automated software for the determination of the first derivative was used according to the manufacturer’s instructions. TEG analysis was performed on citrated whole blood within 2 h of blood collection that was kept at room temperature. For each TEG assay, citrated whole blood (1 mL) was pipetted into a phial containing 1% kaolin and inverted five times to ensure that kaolin was mixed with the blood. Then, 340 µL of kaolin-activated citrated whole blood was transferred to a TEG cup to which 20 µL of 0.2 mol/L CaCl_2_ had been preloaded for re-calcification. The TEG analyzer was stopped 40–60 min after reaching MA at 37 °C. The following five graph parameters were measured when the TEG was complete: (1) reaction time (R) – time from the start of the test to a TEG amplitude of 2 mm, reflecting the combined effect of the coagulation factors involved in the initiation of hemostasis; (2) Kinetic (K)-time: the period from the TEG amplitude of 2 mm to when the curve reached an amplitude of 20 mm, which measured the rate of clot formation (fibrin cross-linking); (3) α-angle: the angle between the tangent line (drawn from the split point to the curve) and the horizontal base line, representing the acceleration of fibrin build-up and cross-linking; the K-time and α-angle thus reflected how fast the clot strength was increasing once clotting began (the levels of circulating fibrinogen); and (4) the MA – indicative of the maximum attainable clot strength that reflected the cross interaction between platelet functions and coagulation. Lysis (LY) of the clot then began; (5) the amount of clot that had dissipated at 30 min was expressed as LY-30 [34].

Normal values for R-time were defined between 5.0 and 10.0 min.
Normal values for K-time were defined between 1.0 and 3.0 min.
Normal values for α-angle were defined between 53 and 72 degrees.
Normal values for MA were defined between 50 and 70 mm.
Normal values for LY-30 were defined between 0% and 3%.
TEG was performed <48 h before biopsy.

This study was approved by the local Institutional Review Board (approval number 0236-18-TLV).

### Statistical analysis

All continuous variables are displayed as means (standard deviation, SD) for normally distributed variables or median [interquartile range (IQR)] for variables with abnormal distribution. Categorical variables are displayed as numbers (%) of patients within each group. The different biomarkers were compared by a Student’s *t* test for normally distributed variables and by the Mann–Whitney *U*-test for abnormally distributed variables. Categorical variables were compared using a χ2 test. Multivariate regression analysis was used to assess the effect of the tested covariates on the different TEG abnormalities and on risk of bleeding.

Two-tailed *p*-values of <0.05 were considered statistically significant. The IBM SPSS Statistics 24.0 statistical package (IBM Corp., Armonk, NY, USA) was used to perform all statistical analyses.

## Results

Percutaneous native kidney biopsies were performed in 417 adult patients: 187 during period A (92 in 2015 and 95 in 2016), and 230 during period B (143 in 2017 and 87 in 2018).

Two hundred and seventy-three biopsies were performed as ambulatory procedures and 144 during hospitalization. Most biopsies (90.6%) were performed by the same radiologist, S.M.

Patients’ mean age at the time of biopsy was 50.5 ± 18.8 years (range 18–91.5). Systolic blood pressure was >170 mmHg in 4.2%. Mean serum creatinine levels were 2.19 ± 2.4 mg/dL. Twenty-two patients had platelet count <100 × 10^9^/L before biopsy, and 14 had abnormal PT and/or aPTT.

Fifty-one of the 187 (27.3%) patients who underwent kidney biopsy during period A, had a BT test, which was normal (<6 min) in all tested patients.

Seventy-one of the 230 kidney biopsy patients (30.9%) during period B underwent TEG before biopsy. Patients were divided between those who were not tested at all, and those who underwent a TEG or BT test before biopsy, according to their baseline characteristics (Table 1). Patients who underwent TEG or BT tests were older, had higher mean serum creatinine and lower mean eGFR, hemoglobin and hematocrit pre-biopsy, while systolic and diastolic blood pressure, platelet count, PT and aPTT did not differ between the groups. There was a similar rate of patients treated with dialysis (all hemodialysis) in groups BT and TEG, before biopsy.

**Table 1.** Patient baseline characteristics before kidney biopsy.

Parameter	No TEG /BT*	TEG**	BT***	*p* Value
Number	305	71	51	
Age (years)	50.1 ± 18.3	55.1 ± 19	61.7 ± 16.6	0.001
Gender, females (%)	42.3	39.1	45.9	0.54
Serum creatinine (mg/dL)	1.3 ± 0.9	2.7 ± 2.1	2.8 ± 2.3	0.004
eGFR (mL/min/1.73m^2^)	69.4 ± 18.3	32.1 ± 20.6	30.8 ± 23.1	0.003
Number of patients on dialysis before biopsy (%)	0 (0)	4 (5.6)	4 (7.8)	0.004
Hemoglobin (g/dL)	12.9 ± 2.7	10.8 ± 2.3	11.1 ± 2.1	<0.001
Hematocrit (g/dL)	38.5 ± 6.1	32.0 ± 7.1	33.1 ± 8.1	<0.001
Platelet count (×10^9^/L)	243.5 ± 90.9	235.5 ± 106	241.6 ± 74.8	0.6
Serum albumin (g/L)	34.4 ± 6.5	33.5 ± 8	33.1 ± 7.1	0.76
aPTT (s)	34.4 ± 6.5	33.5 ± 8	33.1 ± 7.1	0.76
PT (s)	12.6 ± 7.2	11.3 ± 1.9	11.9 ± 2.4	0.156
Systolic blood pressure (mmHg)	143.1 ± 21.9	143.9 ± 22.0	141.7 ± 19.7	0.81
Diastolic blood pressure (mmHg)	86.5 ± 13.2	83.9 ± 15.3	82.9 ± 14.7	0.25
Abnormal Platelet (%)	4.5	7	6.5	0.528
Abnormal aPTT (%)	6.5	1.4	4.6	0.097
Abnormal PT (%)	0.5	0	0.8	0.46
Diagnosis				0.160
Glomerular	49	47	51.2	
Diabetic nephropathy/nephrosclerosis	12.3	32	19.7	

^*^ No TEG or BT tests were done before kidney biopsy.

^**^ TEG test was performed before biopsy.

^***^ BT was performed before biopsy.

aPTT: activated partial thromboplastin time; BT: bleeding time; eGFR: estimated glomerular filtration rate; PT: prothrombin time; TEG: thromboelastography.

### TEG abnormalities

**Figure 1.**
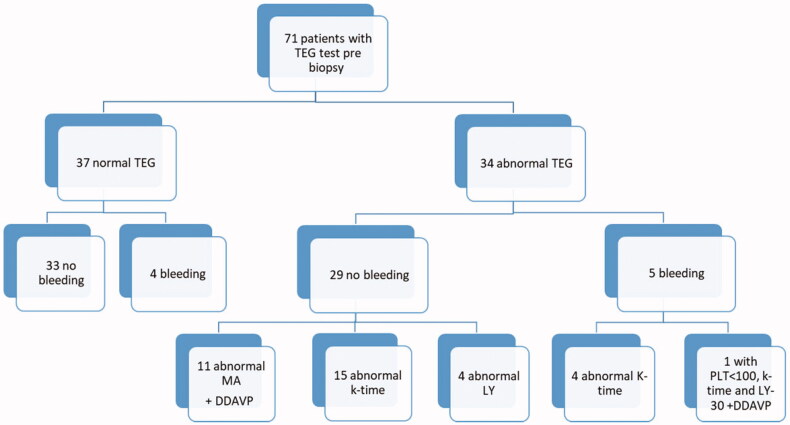
TEG test results before native kidney biopsy, and bleeding complication post biopsy, in 71 patients with serum creatinine above 1.5 mg/dL.

TEG was abnormal in 34/71 patients (Figure 1). All TEG abnormalities pointed toward a hypocoagulability state. Most patients with an abnormal TEG had a combination of abnormal parameters:

- All patients had normal R-time.
- Abnormal MA (<50 mm) was found in 11 patients, of whom 5 also had abnormal LY, and angle and/or kinetics, and 2 of them had abnormal angle and/or kinetics.
- Abnormal LY (>3.0%) was found in 8 patients, of whom 5 also had abnormal MA and angle and/or kinetics, and 3 had abnormal angle and kinetics.
- Abnormal kinetics (>3 min) was found in 3 patients, abnormal α-angle (<53 degrees) was found in 22 patients, and 7 had both abnormalities. Of these 32 patients, 2 also had abnormal MA, 3 had abnormal LY, and 5 others had abnormal MA and LY.

Table 2 shows characteristics of patients according to different abnormal parameters of TEG compared to patients with normal TEG. Patients with TEG abnormalities were older, and patients with abnormal MA had lower mean hemoglobin and hematocrit levels pre-biopsy.

**Table 2.** Characteristics of patients with normal TEG, compared to patients with abnormal MA, K-time and/or α-angle, and LY-30.

	Normal TEG	Abnormal MA	*p* Value	Abnormal K-time/ α-angle	*p* Value	Abnormal LY-30	*p* Value
Number	37	11		32		8	
Other TEG abnormalities (%)		7 (63.6)		10 (31.3)		8 (100)	
Age (years)	48.3 (30.9–63.2)	65.3 (63.4–71.9)	0.003	64.5 (48.2–72.7)	0.005	64.9 (57.9–72.2)	0.020
Gender, females (%)	10 (22.8)	4 (36.4)	1	15 (46.8)	0.148	6 (75%)	0.019
Serum creatinine (mg/dL)	2.04 (1.59–3.49)	2.6 (2.3–3.1)	0.569	2.4 (1.58–3.2)	0.364	2.6 (1.6–3.1)	0.885
eGFR (mL/min/1.73m^2^)	43 (19–56)	27 (21–36)	0.471	29 (19–49)	0.581	27 (21–53)	0.711)
Pre-biopsy hemoglobin (g/dL)	11.1 (9.4–13.1)	9.7 (9.1–10.0)	0.008	9.7 (9.1–10.9)	0.215	9.6 (7.9–9.9)	0.274
Pre-biopsy hematocrit (g/dL)	32.5 (28.1–38.5)	29 (26.9–31)	0.009	29.5 (27–33.8)	0.348	28.5 (23.8–30.5)	0.374
Pre-biopsy platelet (×10^9^/L)	212 (168.5–263.5)	272 (162–399)	0.316	239 (177.5–333.2)	0.147	330 (203–423)	0.126
PT (s)	10.8 (10.5–11.2)	11.1 (10.8–11.4)	0.301	11.2 (10.7–11.7)	0.194	11.4 (10.8–12.6)	0.107
aPTT (s)	28.1 (24.3–29.8)	26.5 (22.7–29.8)	0.264	25.6 (23.9–28.7)	0.109	25.5 (21.6–29)	0.223
Systolic blood pressure (mmHg)	144 (129–158)	142 (130.7–155.7)	0.991	136 (129–155)	0.862	146 (128–167)	0.814
Diastolic blood pressure (mmHg)	83 (74–99)	96 (79.5–100)	0.493	80.5 (72–95.7)	0.358	89 (67.9–99.5)	0.784
Diagnosis: Glomerular (%)	20 (55.5)	7 (63.6)	0.736	18 (56.3)	1	5 (62.5)	1

aPTT: activated partial thromboplastin time; BT: bleeding time; eGFR: estimated glomerular filtration rate; MA: maximal amplitude; PT: prothrombin time; TEG: thromboelastography.

**Figure 2.**
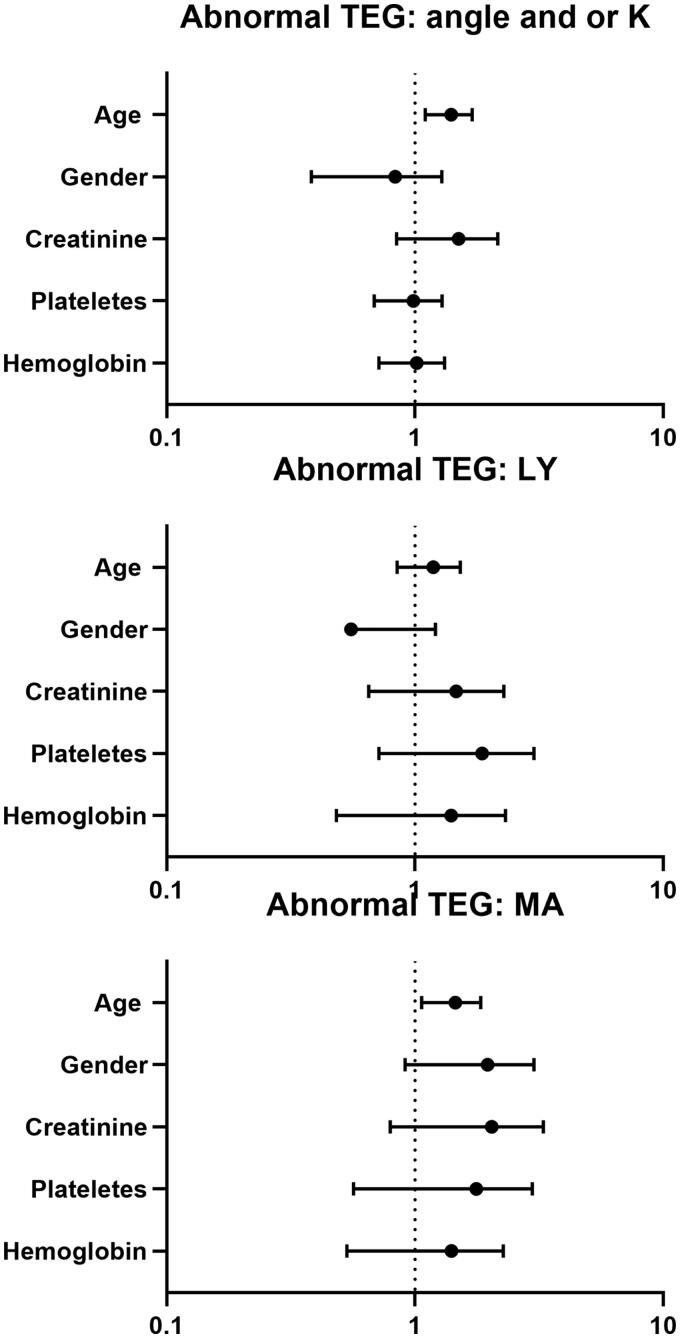
(a–c) Forest plot showing parameters related to risk of developing an abnormal TEG component (divided to abnormal Maximal amplitude (MA); Lysis (LY); Angle and/or Kinetics). Note *x* = 1 (dash line) represents similar risks (odds ratio).

Next, we performed a regression analysis to estimate the effect of the tested covariates on the different TEG parameters (Figure 2(a–c)). The only parameter related to an abnormal MA and K-time/α-angle was older age.

### Post-kidney biopsy bleeding

While mean hemoglobin was 11.69 ± 2.7 g/dL at the time of biopsy, it decreased to 10.4 ± 2.3 g/dL at post biopsy. The mean difference between pre- and post-biopsy hemoglobin was 0.68 ± 0.9 g/dL (*p* = 0.001).

Overall, 26 patients (6.23%) had bleeding complications: 17 had decreased Hb >1.5 g/dl and did not require further intervention; 7 had decreased Hb and hypotension and received at least 1 packed cell transfusion; while 2 others required an angiographic study in addition to a blood transfusion.

During period B, 12 patients had bleeding complications (5.2%), while 14 patients had bleeding complications (7.4%) during period A, *p* = 0.461.

**Figure 3.**
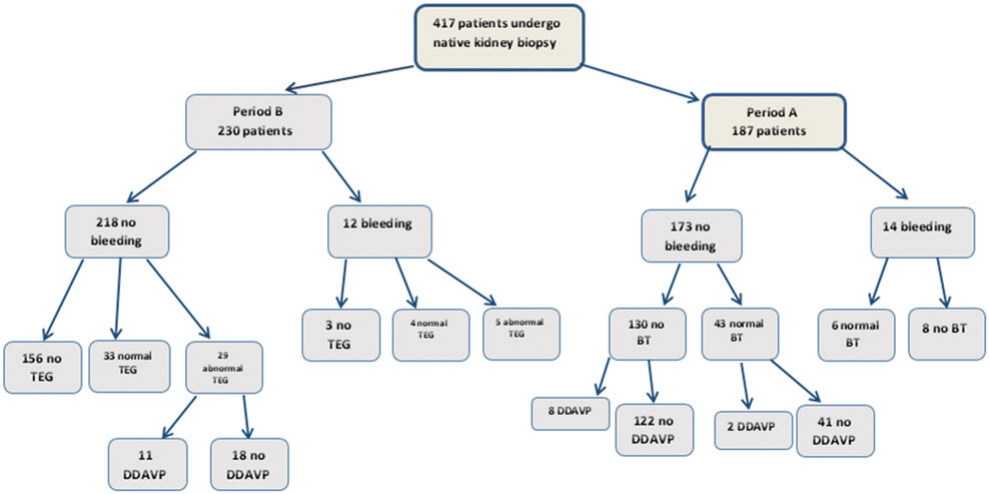
A flow chart of the entire study cohort, in period A and period B, according to patients with and without bleeding complication post kidney biopsy, normal and abnormal TEG or BT test results, and treatment with DDAVP before biopsy.

Figure 3 presents a flow chart of bleeding complications in the entire study cohort. In period B, 5 of 12 patients who bled had an abnormal TEG prior to biopsy, all had abnormal K-time.

A total of 21 patients received DDAVP before biopsy. In period B, there were 11 patients with an abnormal MA (2 of them were on dialysis before biopsy), and one patient with an abnormal TEG (K-time) and thrombocytopenia (PLT = 84 × 10^9^/L). In period A, the 9 patients with suspected uremic thrombopathy were treated with DDAVP. All had creatinine >4.0 mg/dL, eGFR < 32 mL/min/1.73m^2^, blood urea nitrogen (BUN) >90 mg/dl and normal BT, platelet count, PT and aPTT. 4 of them were on acute hemodialysis prior to biopsy.

None of the patients treated with DDAVP had significant bleeding, except for one patient, who had a low platelet count and abnormal K-time.

When taking into account only those patients who underwent TEG before biopsy, there was no statistical difference in bleeding post biopsy between patients with normal vs abnormal TEG (*p* = 0.728).

**Figure 4.**
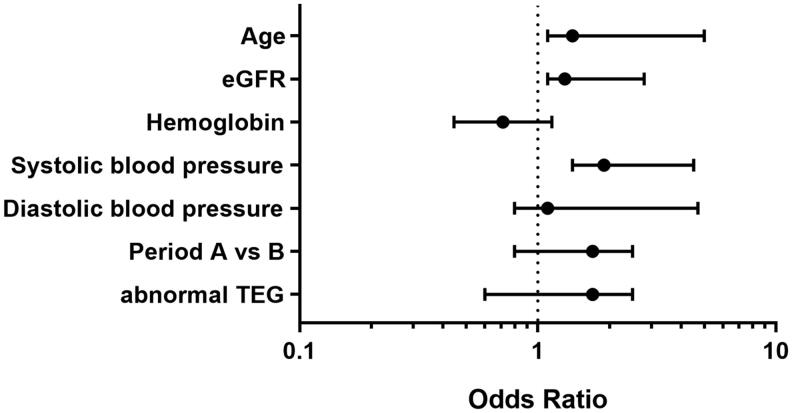
Forest plot showing the parameters related to the risk of bleeding complication post native kidney biopsy. Note *x* = 1 (dash line) represents similar risks for bleeding (odds ratio).

In a multivariate regression model, after excluding patients treated with DDAVP, bleeding complications were significantly related to increased age (Odds Ratio 1.4 [95% CI 1.11–7.48] *p* = 0.04), higher systolic blood pressure (1.85 [1.258–9.65] *p* = 0.02), and increased creatinine (1.21 [1.06–3.134] *p* = 0.048) (Figure 4). There was no significant difference in the risk of bleeding between the two time periods, neither in abnormal nor normal TEG performed before the biopsy. There was also no association between risk of bleeding and etiology of renal failure.

## Discussion

The risk of bleeding post renal biopsy has led to screening for primary hemostasis before the procedure [10]. A BT test, which considered a standard practice for this assessment in uremic patients [11], is controversial [12–15,22]. A possible alternative for the BT test is TEG. However, only limited data exist regarding TEG in patients with renal dysfunction [32]. In our cohort, which comprised 71 patients with serum creatinine of >1.5 mg/dL, about half of the patients had normal TEG parameters, and most of those with abnormal TEG had combined abnormalities, all pointing to a hypocoagulability state. The only parameter that was related to an abnormal TEG was older age. We failed to characterize a specific pattern of TEG abnormalities in patients with renal failure. The use of TEG has been examined in a few studies with inconsistent results. In a cohort of 120 patients who underwent transplanted kidney biopsy, TEG was related to mild coagulation abnormalities associated with the risk of bleeding; the only abnormalities found were angle and K values [32]. Another prospective observational study demonstrated a hypercoagulable state in uremic patients [35]. In a further study, Darlington *et al* compared TEG of hemodialysis patients to controls, and found that R-time values were higher in end-stage renal disease (ESRD) patients, while there were no differences in MA. Normal manufacturer-defined coagulation and aggregation parameters were present in 31% of ESRD patients compared with 56% of controls [29]. In a recent study on patients with different stages of chronic kidney disease and healthy controls, no significant differences were found in the R, K, MA and α-angle values [36].

The main etiologies for uremic thrombopathy include platelet dysfunction as well as abnormal platelet-endothelial interactions [6], due to both decreased platelet aggregation and impaired platelet adhesiveness. Our study, as well as others, failed to find an association between MA (which indicates platelet function) and kidney function. We suggest that these markers for uremic thrombopathy have failed to produce significant results, and call for further studies in this field.

In our study, all patients with abnormal MA were treated with DDAVP and none of them had bleeding complications. All patients, who underwent TEG before biopsy and had bleeding complications, had abnormal K-time. Both K-time and α-angle measure the speed of clot formation or clot kinetics. The most common abnormality in TEG parameters in our study was in α-angle, with a quarter of the patients demonstrating a combination of K-time and α-angle. None of the patients with these abnormalities (isolated α-angle or combined K-time and α-angle) had bleeding complications.

A prolonged K-time may be related to bleeding [35]. However, the finding of isolated abnormal K-time (with normal α-angle and R-time) is nonspecific and usually does not require intervention [37]. The clinical importance of isolated prolonged K-time before kidney biopsy remains unclear. We suspect that isolated prolonged K-time may be related to a technical/laboratory problem, for example the manual addition of kaolin.

The rate of all bleeding complications post native kidney biopsy reported in the literature is about 5–10% [38,39]. The risk for bleeding increases with hypertension, reduced kidney function, anemia, and older age [38–46]. The risk of bleeding may be decreased by the administration of DDAVP prior to biopsy [47,48]. The bleeding rate in our cohort was relatively low (2.15% of patients required blood products and/or angiographic intervention, and 4.07% had decreased hemoglobin). Our study, in accord with others, found that decreased kidney function, higher systolic blood pressure and older age are related to bleeding. In our center, most native kidney biopsies are performed by the same dedicated experienced radiologist. Thus, this cohort provides us with a unique opportunity to investigate parameters related to bleeding, without the confounding factor of the operator parameter, and allows us to strengthen our conclusions regarding the clinical parameters associated with bleeding.

The main finding in our study was that risk of bleeding was comparable between the two time periods: in period A (2015–2016), patients with a suspected tendency toward uremic bleeding were treated with DDAVP, while their laboratory evaluation (BT) was normal. In period B (2017–2018) patients underwent TEG and were treated with DDAVP when the MA component was low. Our data suggest that performing TEG prior to a kidney biopsy does not provide any benefit over clinical judgment. Therefore, we challenge the practice of performing a BT test and TEG prior to a kidney biopsy, and prefer to treat empirically with DDAVP, based on kidney function and/or clinical parameters (for example bleeding diathesis).

Our study has a few limitations. First, this is a retrospective analysis and is prone to flaws related to the study design. Second, the number of bleeding events was relatively low. An explanation for the low rate might be the experience of a single biopsy operator. Nevertheless, it allowed us to explore the procedural risk without being confounded by the operator parameter. Third, the number of patients with a pre-biopsy TEG was relatively low (*n* = 71), and included only patients with renal dysfunction, without a control group of patients with normal kidney function. A fourth limitation is the bias in including patients with abnormal MA who were treated prophylactically with DDAVP without an appropriate control group. A fifth limitation is a potential bias of excluding patients treated prophylactically with DDAVP from the regression model analyzing parameters related to risk of bleeding post biopsy. And another limitation is the difference between the groups of patients underwent BT and TEG tests; however, the statistical analysis was adjusted for confounders including age.

In conclusion, we found that TEG abnormalities in patients with renal dysfunction are variable and fail to predict bleeding during kidney biopsy. The decision to administer DDAVP as a preventive measure during these procedures should be based on clinical judgment only.
